# A high-density EEG study of differences between three high speeds of simulated forward motion from optic flow in adult participants

**DOI:** 10.3389/fnsys.2015.00146

**Published:** 2015-10-26

**Authors:** Kenneth Vilhelmsen, F. R. (Ruud) van der Weel, Audrey L. H. van der Meer

**Affiliations:** Developmental Neuroscience Laboratory, Department of Psychology, Norwegian University of Science and TechnologyTrondheim, Norway

**Keywords:** EEG, optic flow, motion perception, speed differences, adults, visual evoked potential (VEP), time-frequency analysis, temporal spectral evolution (TSE)

## Abstract

A high-density EEG study was conducted to investigate evoked and oscillatory brain activity in response to high speeds of simulated forward motion. Participants were shown an optic flow pattern consisting of a virtual road with moving poles at either side of it, simulating structured forward motion at different driving speeds (25, 50, and 75 km/h) with a static control condition between each motion condition. Significant differences in N2 latencies and peak amplitudes between the three speeds of visual motion were found in parietal channels of interest P3 and P4. As motion speed increased, peak latency increased while peak amplitude decreased which might indicate that higher driving speeds are perceived as more demanding resulting in longer latencies, and as fewer neurons in the motion sensitive areas of the adult brain appear to be attuned to such high visual speeds this could explain the observed inverse relationship between speed and amplitude. In addition, significant differences between alpha de-synchronizations for forward motion and alpha synchronizations in the static condition were found in the parietal midline (PM) source. It was suggested that the alpha de-synchronizations reflect an activated state related to the visual processing of simulated forward motion, whereas the alpha synchronizations in response to the static condition reflect a deactivated resting period.

## Introduction

The information contained in the optic flow field is thought to be sufficient for maneuvering in the environment, and several studies have shown that optic flow information gives vital clues about direction (Warren et al., 2001; Bruggeman et al., 2007). Over the last few decades numerous studies on both optic flow and visual motion in general have gathered a large amount of data on how this kind of information is perceived. However, some questions still remain regarding how naturalistic stimuli are perceived. We constantly move around in an environment that changes according to our movements, and the optic flow field provides detailed information about our position in the environment and where we are heading (Bruggeman et al., 2007; Bruggeman and Warren, 2010). When we increase our velocity the environment might be static, but not our position relative to it and the question then arises as to how we perceive the changing velocity in the optic flow field. Optic flow is the sum of all the information contained in the visual array, resulting from the information contained in invariants in the environment created by motion (Gibson, 1966). Velocity changes in the optic flow field would most likely be reflected in changes in brain activity. So the functional relevance of optic flow becomes apparent as it is an indicator of our motion through a three-dimensional world. Optic flow fields contain information about the relative movements of objects in relation to the observer, thus simulation of optic flow more closely represents real-world motion.

Motion processing has been found to occur predominantly in the V5/MT area of the human brain (Zeki et al., 1991; Holliday and Meese, 2008; Hayashi et al., 2010; Basso et al., 2012; Pitzalis et al., 2012; Vetter et al., 2013). Several optic flow studies have been conducted to investigate the neurological basis for visual motion perception and to study the brain structures involved in this process, and the V5/MT and MST areas have been identified as being especially sensitive to optic flow (Duffy and Wurtz, 1997; Duffy, 1998; Morrone et al., 2000; Holliday and Meese, 2005, 2008; Smith et al., 2006). It has also been shown in both human and animal studies that area 7a, the right latero-posterior precuneus and the motor cortex, above V5/MT and MST, are involved in motion perception, including optic flow (Phinney and Siegel, 2000; Merchant et al., 2001, 2004; Liu and Newsome, 2003). A large number of neurons in rhesus monkey MST are sensitive to a speed gradient stimulus, where the speed is slower in the center of the stimulus, and increases toward the periphery (Duffy and Wurtz, 1997). Duffy and Wurtz (1997) also found a difference between non-gradient and gradient stimuli, showing that the MST area is specialized for processing optic flow patterns. This was also observed in a study by Liu and Newsome (2005) where, by investigating single cells and multi-unit clusters in the macaque monkey, activation in the MT area was reported. There seems to be sensitivity to ecological motion in the MT/V5 and MST areas, although these areas also respond to translational and non-gradient visual motion.

Vection studies have investigated simulated motion and optic flow. Vection is defined as the subjective experience of self-motion as a result of visual, auditory or haptic information, and optic flow has been shown to elicit a sensation of vection (Palmisano et al., 2015). Visual motion stimuli that induce vection elicited significantly more activity in areas such as MT+, M6, VIP, and PIVC, compared to when participants did not experience vection (Uesaki and Ashida, 2015). This indicates that some brain areas respond stronger in response to vection. Removal of optic flow in normal walking has been shown to reduce subsequent vection as well (Seno et al., 2015). It has also been argued that the most important contributor to vection is stimulus depth (Palmisano et al., 2015), indicating that perception of optic flow contributes to the genesis of vection.

van der Meer et al. (2008) conducted a high-density EEG study to investigate infants' and adults' brain responses comparing structured forward optic flow to random visual motion. They found latency differences between adults and infants, where infants seemed to process information slower, as shown by higher N2 latencies. Interestingly, both groups showed shorter N2 latencies for structured optic flow compared to random motion, and this was interpreted to be a result of more coherence in, or reflect the higher importance of, structured motion for both infants and adults. Agyei et al. (2015) also found, using similar optic flow stimuli, that 12-month-old infants showed shorter latencies for forward optic flow compared to reversed and random motion.

The amplitude and latency of the N2 component have also been linked to the speed of motion, and a decrease in latency and an increase in amplitude for increasing speed is the most common finding (Heinrich, 2007). Maruyama et al. (2002) reported this using a random dot kinematograph and relatively low motion speeds of up to 20°/s in a MEG study. Another MEG study conducted by Kawakami et al. (2002) using a much wider range of speeds reported the same results, but when speed increased to above 100°/s, latency increased with speed of motion.

Keshavarz and Berti (2014) reported that a translational stimulus with a stationary center and a moving periphery showed higher amplitude in the N2 component in channels O1 and O2, and participants reported a strong sensation of vection in response to this condition in a test performed after the EEG recordings. The other conditions were moving center and stationary periphery, stationary center and periphery, and moving center and periphery. They argued that the component could be the initial step in the generation of vection as the presentation of the stimuli was too short (2.5–3.5 s) to induce vection. It was also suggested that integration of visual information triggered the perception of vection.

Recently, there has been more and more focus on analyzing oscillatory EEG activity. Motion-induced power increases in gamma-band (30–80 Hz) activity (Hoogenboom et al., 2006) and beta-band (14–30 Hz) activity (van der Meer et al., 2008) over occipital electrodes in adults have been reported. In connection with visual stimulation, alpha-band oscillations are linked to either an attentive or an idling state. Schürmann et al. (1997) used simple light onset visual stimuli and found alpha oscillations in the occipital cortex. They argued that occipital alpha oscillations are related to primary sensory perception, while slower theta oscillations are responses to cognitive or associative processes. In several studies these alpha oscillations have been reported in connection with visual stimulation (Pfurtscheller et al., 1994; Niedermeyer, 2005; Hanslmayr et al., 2011; Mathewson et al., 2011).

(Alpha-)synchronizations often reflect an idling state (Pfurtscheller, 1992; Pfurtscheller et al., 1996; Foxe and Snyder, 2011; Mathewson et al., 2011), while a following or earlier de-synchronization reflects an activation period or an attentive state (Pfurtscheller et al., 1994; Klimesch, 1999). It has also been suggested that an increase in alpha power reflects inhibition, whereas a decrease is a result of release of inhibition (Klimesch et al., 2007; Klimesch, 2012). In connection with activation, two separate alpha event-related de-synchronizations (ERDs) are often found: one lower alpha situated over occipital areas and one higher alpha situated over parietal areas. The former has been linked to the processing of visual events, while the latter is thought to be involved in cognitive processes and attention (Pfurtscheller et al., 1994).

The present study investigated, using high-density EEG, how occipital and parietal areas respond to different speeds simulating forward motion in an optic flow paradigm. So as to create a realistic scene, a novel motion stimulus was used, where participants were presented with approaching poles on each side of the visual field, simulating forward motion down a road at three different driving speeds. This was different from several previous EEG studies on velocity (e.g., Kawakami et al., 2002; Maruyama et al., 2002) that have studied object motion. Visual components in the corresponding visual evoked potentials (VEPs) were analyzed and it was investigated whether, and if so how, speed differences affect induced non-phase-locked changes in the time-frequency domain. By understanding how the brain responds to an ecologically plausible stimulus moving at different speeds, we might come closer to understanding how real world events, and speed changes, are processed by the motion sensitive areas of the brain.

## Materials and methods

### Participants

Participants were recruited from the Dragvoll campus, NTNU (Trondheim, Norway). Twelve participants were tested (7 males), between 18 and 33 years of age (Mean = 25 years, *SD* = 3.8). All participants were right-handed, and had normal or corrected-to-normal vision (one participant wore glasses during the experiment). Two male participants could not be included in the analyses due to excessive noise in the data, resulting in 10 participants whose data were analyzed.

Before signing a consent form, participants were given an information letter explaining the purpose of the study and they were informed that they could at any time and without consequence abort the experiment. This study has been approved by the Central Regional Committee for Medical and Health Research Ethics (REC Central) and the Norwegian Social Science Data Services (NSD).

### Stimuli

The optic flow pattern was generated by E-Prime software (Psychological Software Tools, Inc.) and mirror-projected onto a screen (1.08 × 0.705 m) by an ASK M2 projector with a refresh rate of 60 Hz. The screen had a resolution of 593 px/m. The participants were seated approximately 75 cm from the screen so that the screen subtended an angle of 71.5° by 50.4°.

**Figure 1:**
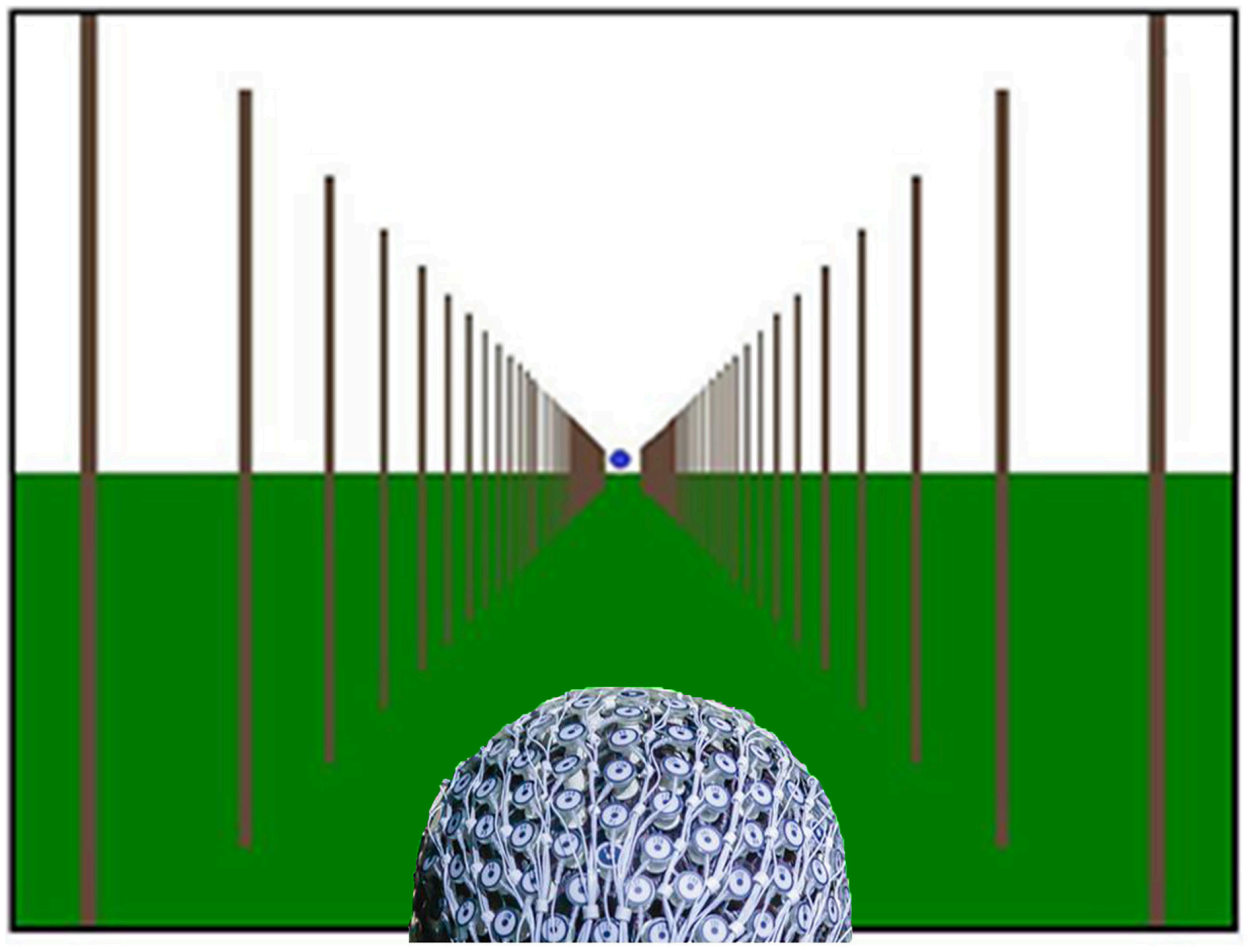
**Optic flow pattern simulating forward motion down a virtual road with a subject wearing the Geodesic Sensor Net**. Blue dot in the center of the screen represents gaze data and indicates where participants were instructed to look, but was not present during the experiment (Supplementary Video 1).

Vehicle driving at three different speeds was simulated by showing forward motion from optic flow on the screen. The three different speeds were set to 25, 50, and 75 km/h, and are referred to as low, medium and fast speed, respectively. Participants were shown several poles, which started from the center of the screen and traveled outwards, leaving the screen at each side, which simulated forward motion (Figure 1, and Supplementary Video 1). The visual angles, defined by the first (road end) and last (road start) position of the poles, resulted in a road length of 30 m.

So as to prevent motion adaptation, a static control condition was shown after every forward motion condition. This inter-stimulus interval (ISI) was created by showing a static stimulus consisting of the same number of poles as in the preceding trial. This was done to avoid any difference in luminance between the forward motion conditions and the static condition.

The forward motion trials lasted 1000 ms each, while the static control trials lasted 3000 ms, giving a duty-cycle of 3:1. There were 75 trials for each of the three forward motion conditions, and 225 trials for the static condition, so the experiment took a total of 15 min.

All participants were monitored via two digital video cameras, and five participants had their gaze monitored by a Tobii X50 camera, but gaze data were not used for further analysis. It was confirmed that participants were able to hold their gaze firmly fixed on the center of the screen and very seldom moved their eyes. Additional visual inspection confirmed that the participants did not produce severe eye movements, and following artifact correction showed no severe horizontal or vertical eye movements. In general, the participants were able to sit still and they performed the task without problems.

This study used chromatic stimuli with a green ground and brown poles on a white background (top half of the screen). The speed of the poles had to be kept below approximately 100 km/h because at these speeds participants in a pilot study reported a reversal and momentary standstill of the poles, a phenomenon known to be a result of spatial under-sampling (Wang et al., 1996). None of the participants reported this effect in the current study, indicating that we successfully managed to avoid it.

### Apparatus

Electroencephalogram (EEG) activity was recorded using a high-density Geodesic Sensor Net 200 (GSN; Tucker, 1993). The net consisted of 256 Ag/AgCl sponge sensors, and three different sized nets (small, medium, and large) were used depending on the participant's head size. Net Station software on a Macintosh computer recorded amplified EEG signals. Onset and offset triggers were created by the E-Prime tool and transferred to NetStation. Impedance was kept below 50 kΩ as recommended for high-input-impedance EGI amplifiers (Picton et al., 2000; Ferree et al., 2001). Data were recorded with an online filter of 0.1 Hz low cut-off (high-pass) and 200 Hz high cut-off (low-pass), with a sampling rate of 500 Hz. Data were stored and transferred to another server for off-line analysis. The Cz electrode was used as reference electrode during the recordings.

### Procedure

When a participant arrived he/she was given the information letter and signed the consent form. The EEG net was prepared in electrolyte solution to ensure good impedance. After the net was mounted the participant was led into the experimental room. The participant was seated in front of large screen in a dimly lit room and was told to focus on the middle of the screen (see Figure 1) as if he/she was driving a car, while being aware of the three different speeds of forward motion. These instructions were given to ensure that the participant was focused on the task at hand. This focus at the center helps produce the sensation of moving through an environment as would be expected in global motion, and participant eye gaze was verified during offline analyses as well.

### Brain data analyses

VEP and time-frequency analyses were carried out in BESA 5.3 (Brain Electrical Source Analysis, BESA GmbH). For the VEP analysis the data were divided into epochs of −300 to 600 ms, and pre-stimulus baseline was from −300 to 0 ms. Artifact correction was performed semi-automatically to correct for physiological artifacts caused by eye blinks or eye movements (Ille et al., 2002). All trials and epochs with amplitudes over 200 μV, gradients over 75 μV/sample and signals below 0.1 μV were left out of further analysis. Bad channels were discarded, but no more than 10% (24 channels) in each participant (no more than 5 electrodes in parietal and occipital areas, and no two electrodes adjacent to each other were removed). Average trial contribution for all participants were (out of 75) 71 for low speed (SD: 3.8), 73 for medium speed (SD: 1.5), 71 for high speed (SD: 3.0), and (out of 225) 216 for the static control condition (SD: 6.6).

### VEP analysis

Data were recorded with a GSN net consisting of 256 channels that were positioned according to the extended international 10–20 system. The high-density channels allow for adequate spatial sampling of information. This was later exported into the standardized 81-electrode configuration of the international 10–10 system for the VEP analysis. The signal for the reference free (10–10) montage was estimated with the spherical spline interpolation (Perrin et al., 1989). Furthermore, an approximated signal on a new virtual electrode should be similar to the signals of the measured electrodes which are spatial neighbors of the estimated one. Filters were set to 1.6 Hz low cut-off (high band-pass) to remove slow drift in the data, and 30 Hz high-cut off (low band-pass). Notch filter was never changed from 50 Hz and was always on. The individual averages were combined into a grand average, and used as a reference for selecting the individual N2 components. The N2 components of the individual averages were then identified using 3D spherical spline whole-head voltage maps identifying maximum N2 activity over parietal areas for the most dominant waveform. Latencies and amplitudes were recorded and subjected to further analysis. Peak latency was measured from stimulus onset to the peak of each N2 component and peak amplitude recorded relative to pre-stimulus baseline.

### Time-frequency analysis

**Figure 2:**
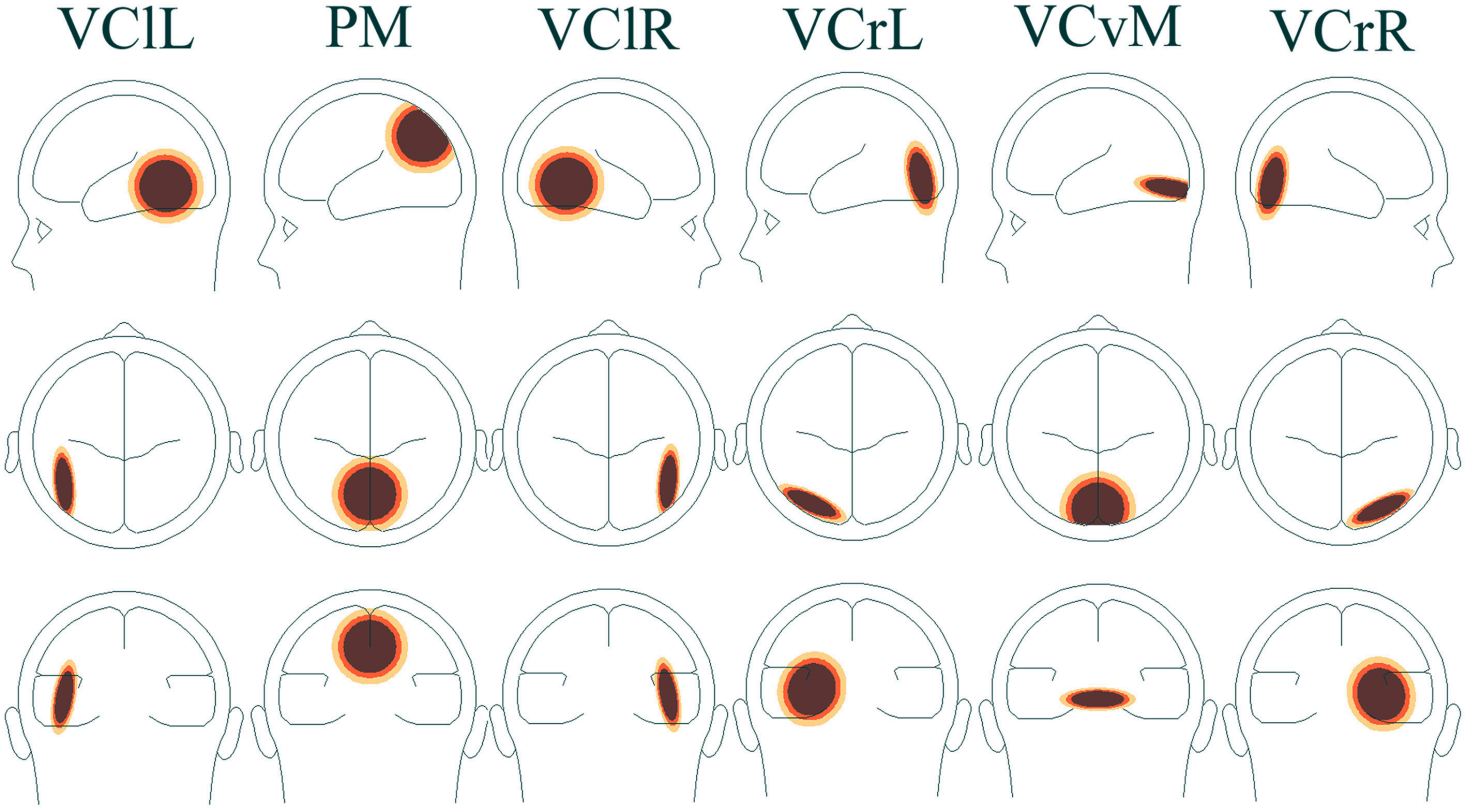
**Head model of associated brain regions in visual cortical areas VClL, VClR, VCrL, VCvM, VCrR, and visual parietal area PM**.

In addition to peak analysis of VEP components, a time-frequency analysis was carried out. The time-domain signal was transformed into the time-frequency domain by complex de-modulation (Papp and Ktonas, 1976). A pre-defined four-shell ellipsoidal head model (Berg and Scherg, 1994; Hoechstetter et al., 2004) was applied and used to transform data from electrode level to source montage dipoles. The wide distribution of focal brain activity at scalp surfaces due to the nature of dipole fields and the smearing effect of volume conduction in EEG means that scalp waveforms have mixed contributions from underlying brain sources and, as a result, measuring oscillatory activity on scalp surface electrodes may not be ideal. Optimal separation of brain activity was therefore achieved using source montages derived from a multiple source model where source waveforms separated different brain activities (Scherg and Berg, 1991). A VEP montage was used, which has a higher number of sources mapping the visual area. Sources included in the analysis were [with Talairach coordinates (Talairach and Tournoux, 1988)]: visual cortex lateral left (VClL), *x* = −45.2, *y* = −57.2, *z* = 6.5, visual cortex lateral right (VClR), *x* = 45.2, *y* = −57.2, *z* = 6.5, parietal midline (PM), *x* = 0.0, *y* = −72.3, *z* = 37.0, visual cortex radial left (VCrL), *x* = −25.6, *y* = −73.0, *z* = 4.2, visual cortex vertical midline (VCvM), *x* = 0.0, *y* = −84.9, *z* = −14.3, and visual cortex radial right (VCrR), *x* = 25.6, *y* = −73.0, *z* = 4.2 (Figure 2).

Time-spectrum evolution (TSE) displays were set from 2 to 30 Hz, and frequency and time sample were set at 1 Hz and 50 ms, while filters were kept at 1.6 Hz low cut-off (high-pass) and 30 Hz high cut-off (low-pass). Averaged waveforms were removed, and power (μV^2^) was used as the measure unit. The static inter-stimulus interval was used as a control and was also computed separately. For the time-frequency analysis the epoch was set from −100 to 1000 ms. A bootstrapping method was performed in each TSE plot for each of the participants to test for significance, and significance level α was set to 0.05.

Paired samples *t*-tests were conducted in BESA Statistics 1.0 (BESA, GmbH) to test for significant differences between conditions in the time-frequency domain. In this analysis the TSE data from all participants were averaged and analyzed together. BESA Statistics uses a combination of permutation testing and data clustering (Bullmore et al., 1999; Ernst, 2004; Maris and Oostenveld, 2007) to avoid the multiple comparisons problem. First, data clusters that show a significant effect between conditions are defined. Then, the clusters are put through several permutations, and a new *t*-test is computed for each permutation and a new cluster value is found. The significance of the initial cluster value is determined based on this new distribution. If the values of condition 1 are lower than the values of condition 2, a negative cluster is found which shows the direction of the statistical effect. There were 1024 permutations for each *t*-test (low speed-static, medium speed-static, and high speed-static), with frequency ranges and epochs the same as in the time-frequency analysis.

## Results

### VEP analysis

**Figure 3:**
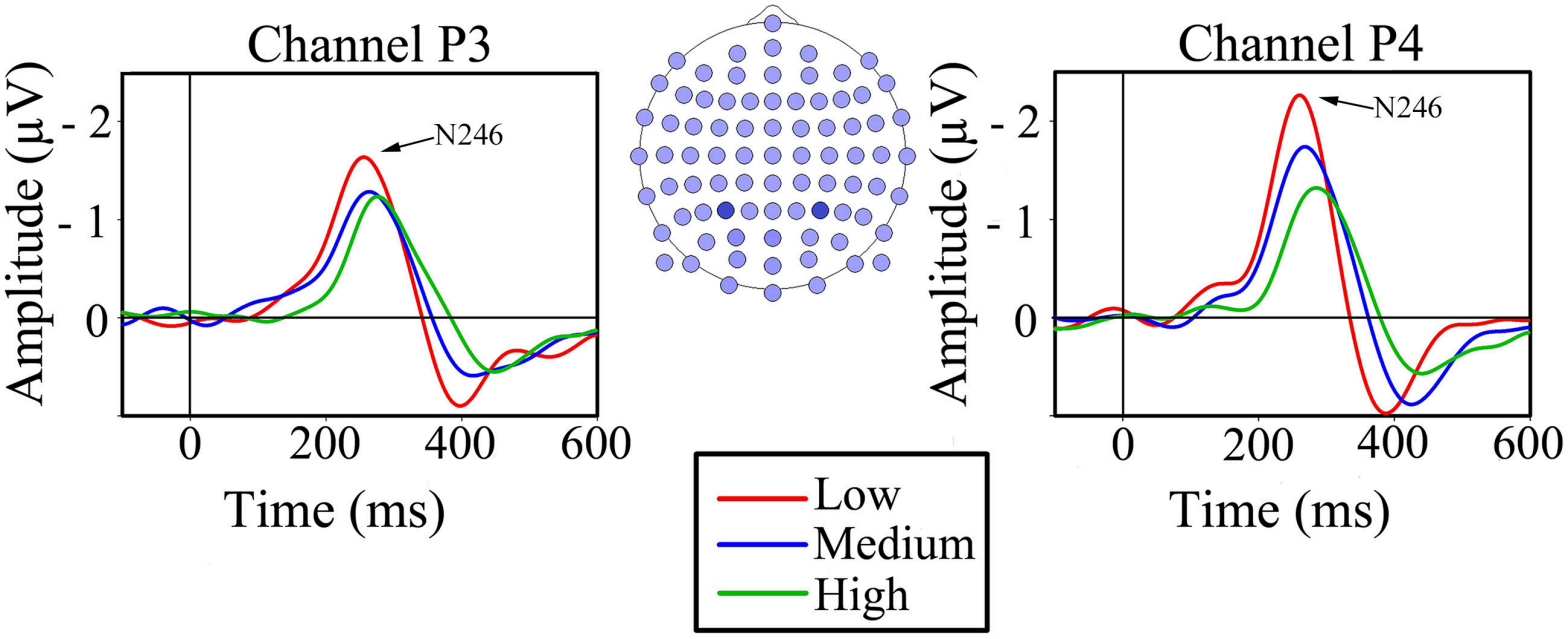
**Grand average VEPs for the three conditions of simulated forward motion in parietal channels P3 and P4**. The head drawing (nose up) shows scalp localization of the 81 standard electrodes with the electrodes of interest indicated with filled blue circles: P3 (left) and P4 (right).

As expected, the N2 component of visual motion was found in all channels in the occipital and parietal areas. However, analyses were carried out on the parietal channels P3 and P4 as they are situated over MT/V5, the area known for processing visual motion and speed differences (Duffy and Wurtz, 1997; Liu and Newsome, 2003; Smith et al., 2006). The N2 component had a latency of 246 ms (*SD* = 36) in parietal channel P3 and 246 ms (*SD* = 30) in parietal channel P4 at low speed, while it was 254 ms (*SD* = 42) and 272 ms (*SD* = 36) at medium speed, and 265 ms (*SD* = 41) and 292 ms (*SD* = 37) ms at high speed (Figure 3).

Two 2-way repeated measures ANOVAs were conducted, with simulated speed (low, medium, and high) and channel (P3 and P4) as within-subjects factors for peak latency and peak amplitude separately. The ANOVA that measured the effect of speed and channel on latency showed a significant main effect of speed, *F*_(2, 18)_ = 36.06, *P* < 0.001, showing that in both channels latency increased as the speed increased (Figure 3). There was no significant main effect of channel, *F*_(1, 9)_ = 1.346, *ns*, but there was a significant interaction effect between speed and channel, *F*_(2, 18)_ = 5.70, *P* < 0.05, indicating that channel P4 had a greater increase in latency with increasing simulated speed than channel P3.

Peak amplitudes in the parietal channels P3 and P4 were −2.63 (*SD* = 1.06) and −3.30 (*SD* = 1.40) μV at low speed, −2.27 (*SD* = 0.97) and −2.38 (*SD* = 0.98) μV at medium speed, and −1.34 (*SD* = 1.68) and −2.05 (*SD* = 1.08) μV at high speed (Figure 3). The ANOVA that studied the effect of speed and channel on peak amplitude yielded a significant main effect of speed, *F*_(2, 18)_ = 10.53, *P* < 0.05, indicating that the amplitude decreased as the simulated speed increased (Figure 3). There was no significant main effect of channel, nor was there a significant interaction between speed and channel.

### Time-frequency analysis

**Figure 4:**
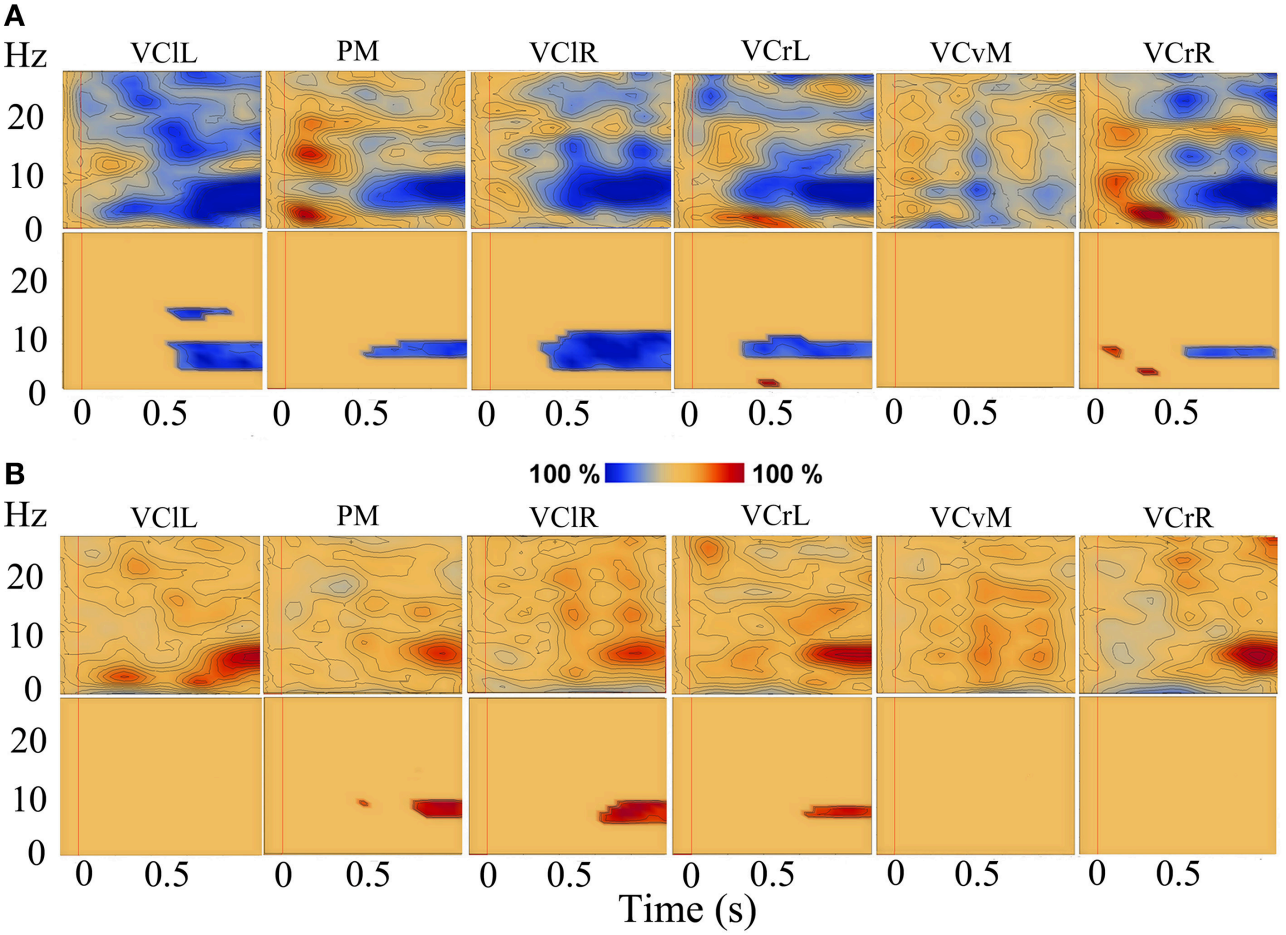
**TSE maps (top) and corresponding TSE probability maps (*P* < 0.05; bottom)**. TSE data in the low speed condition **(A)** and in the static condition **(B)** for a typical participant, in sources (from left to right): VClL, PM, VClR, VCrL, VCvM, and VCrR. Red vertical line at *t* = 0 indicates stimulus onset.

A time-frequency analysis was carried out for the three forward motion conditions and the static condition for all participants individually. Figures 4A,B show TSE maps (top panels) and TSE probability maps (bottom panels) for one typical participant for the low speed motion condition and the static condition, respectively. For all participants, and most consistently so in the parietal midline (PM) source, the three speeds of forward motion showed induced alpha-band de-synchronization from 400 ms to and beyond stimulus end, and the comparisons in the TSE probability maps showed similar results, i.e., a significant decrease in alpha oscillations (see Figure 4A). In the static condition, the opposite induced synchronization in the alpha-band was found, and the corresponding TSE probability maps showed a significant increase in alpha synchronization as compared to the baseline (see Figure 4B).

**Figure 5:**
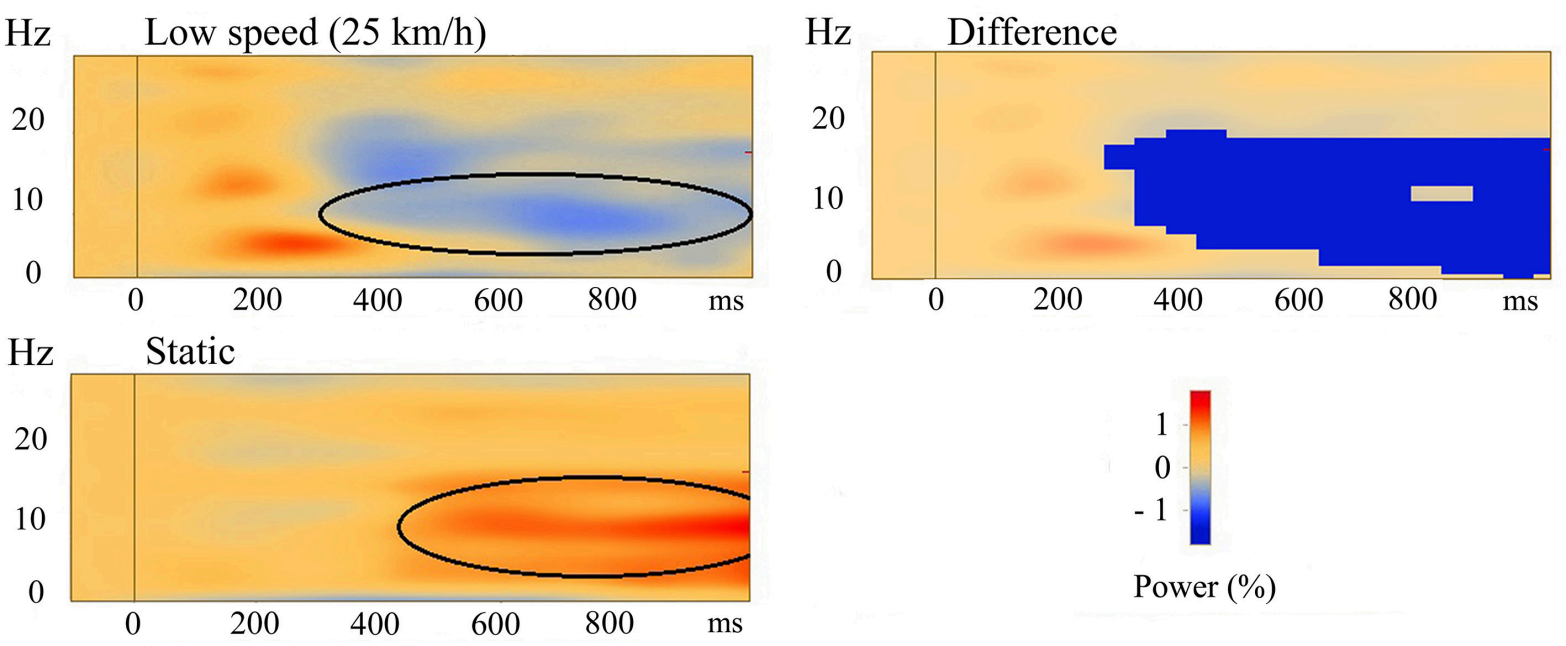
**Averaged TSE plots of all participants in the PM source**. Top left is for the low speed condition, bottom left is for the static condition. Areas of de-synchronization (in blue) and synchronization (in red) are shown. Top right is the area of significant difference (*P* < 0.05) between the low speed and the static condition. The dark blue area indicates that this difference was negative, meaning that the cluster values in the low speed condition (black oval in top left) were significantly lower than the cluster values in the static condition (black oval in bottom left).

The time-frequency analyses from all participants were averaged and the three different motion conditions were compared to each other, as well as to the static control condition. No significant differences between the three speeds were found when they were compared to each other, indicating a similarity in the induced response to visual motion. Then, low, medium, and high speeds were each compared to the static condition. Significant differences were found between the de-synchronization in the alpha-band in the three motion conditions and the synchronization in the alpha-band in the static condition in the PM source. Paired-samples *t*-tests showed significant differences between low speed and the static condition (*P* < 0.05), between medium speed and the static condition (*P* < 0.05), and between high speed and the static condition (*P* < 0.005). Maximum activity was found in the alpha band in the time range from 600 to 800 ms, while there was activation from 300 ms to stimulus end. The significant differences were found in the frequency ranges between 5 and 17 Hz in time ranges from approximately 300 ms to stimulus end in all conditions (see Figure 5 for low speed compared with the static condition), showing that the motion conditions had significantly lower power compared to the static condition, which showed higher power (synchronization) in the PM source.

## Discussion

This study was conducted to investigate brain responses to high speeds of simulated forward motion from optic flow. The stimulus was a road simulated by poles moving from near the center of the screen and out toward the edges of the screen, creating a realistic simulation of an optic flow field. Scalp potentials in two parietal channels of interest were investigated for the three different driving speeds (low, medium, and high) of forward motion and a time-frequency analysis was performed.

We found significant differences between the three forward motion speeds in parietal channels P3 and P4 in the VEP analyses. Peak latency significantly increased as speed increased, while amplitude decreased as speed increased. The time-frequency analysis showed alpha de-synchronizations in response to forward motion, while the static condition showed alpha synchronizations. Alpha de-synchronizations were not significantly different between the three forward motion speeds. The parietal midline (PM) source showed significant differences between alpha de-synchronizations in response to forward motion and alpha synchronizations in response to the static control condition.

Our results, showing that peak latency increased with the speed of forward motion but that peak amplitude was inversely related to the speed, are different from the findings in a study using considerably lower speeds (Maruyama et al., 2002), and are the opposite of what Heinrich (2007) in a review article concluded to be the most common finding. However, for high speeds of motion there are a number of studies that corroborate the present findings. An MEG study measuring neural responses to light spot motion onset with a wide range of motion speeds, reported a decrease and subsequent increase in latencies as a function of speed of motion (Kawakami et al., 2002). In another study, Maunsell and Van Essen (1983) reported that in the macaque monkey, most speed sensitive neurons in the MT area have a preference for relatively low motion speeds. Thus, with fewer neurons preferring higher speeds, peak amplitude is likely to decrease with motion speed at the high end of the scale. Amplitude reflects the number of synchronously active neurons (Elul, 1972; Pfurtscheller and Lopes da Silva, 1999). Low amplitude indicates fewer neurons firing in synchrony, and therefore fewer neurons attuned to the particular condition. Kawakami et al. (2002) argued that amplitude change is related to the size of the neuronal population responding to the stimuli. The present findings showed that amplitude decreased with increasing speed of forward motion, suggesting that most of the neurons in the motion sensitive area were attuned to the lowest speed. Indeed, Liu and Newsome (2003) found that neurons are clustered according to preference of speed. Thus, these studies together with the present findings provide evidence that motion speeds over a certain magnitude give rise to increasing latencies and decreasing amplitudes.

Earlier studies on visual motion perception with adults have generally reported N2 latencies of approximately 150–250 ms (Kuba and Kubová, 1992; van der Meer et al., 2008), while this study reported latencies of up to 292 ms. These longer latencies could indicate that the optic flow pattern specifying forward motion at driving speeds used in the present study was more challenging than previously used.

From a life-span developmental perspective, it has been argued that increased latencies in response to visual motion reflect slower information processing (Langrová et al., 2006; van der Meer et al., 2008). The increased latencies in the current study might be due to the increased amount of information contained in the high speed condition and, as a result, the participants might have perceived it as more complex than the lower speeds. This could explain why high speeds result in slower information processing compared to lower speeds. The increased latencies in response to the higher speeds might also reflect that high speeds are less familiar than lower speeds, as humans do not encounter these speeds as often in the real world. A long response time has been argued to be a result of a lack of, or less specialized, neuronal networks (Howard et al., 1996; Johnson, 2000; Dubois et al., 2006) and it has been suggested that an increase in processing speed might be the result of an individual's experience with different environmental stimuli (Mukherjee et al., 2002; Agyei et al., 2015). White matter myelination increases with experience and increases from infancy to adulthood, and improved white matter tracts have been shown to be related to improved processing speed (Mukherjee et al., 2002; Fields, 2008). This might indicate that participants had more experience with the lower speeds which resulted in lower latencies. Demanding tasks are thought to put a greater workload on the system (Strayer and Drews, 2007; Allison and Polich, 2008) and these have been shown to decrease the magnitude of the response. Our findings showed this decrease in amplitude in response to the higher optic flow speeds as compared to the lower speed. This might indicate that the visual system when dealing with higher speeds of simulated forward visual motion is put under greater strain and, therefore, displays lower amplitudes. The higher optic flow density at the higher speeds of simulated forward visual motion might be more demanding for the visual system.

Latencies in channel P4 showed a significantly greater increase with motion speed compared to channel P3. This finding suggests that the right hemisphere is more involved in the processing of optic flow. Right lateralization for motion stimuli was also found in a study by de Jong et al. (1994), who found that the right latero-posterior precuneus (superior parietal lobe) was an important contributor to the processing of optic flow, fed by area V3.

The current study found latency and amplitude changes in the N2 component as a function of speed changes in simulated forward visual motion from optic flow. The N2 component has been shown to respond to visual motion stimuli that can induce vection in participants. These N2 components have also been found in response to vection-inducing stimuli as reported by Keshavarz and Berti (2014). They found the highest N2 amplitudes in O1 and O2 in response to a translational moving stimulus with a moving periphery and stationary center. However, the authors stated that the stimuli used were too short (2.5–3.5 s) to induce vection during EEG recordings, but argued that their N2 findings could indicate the initial steps in vection processing. Vection ratings were obtained using longer visual motion presentations (45 s) after the EEG recordings, and the results showed highest vection ratings for the stationary center and moving surround stimulus. A study by Thilo et al. (2003) found a decrease in the N70 component in occipital electrodes O1, Oz, and O2 in response to perceived rotational self-motion with participant reports of vection. These studies have not investigated speed changes in optic flow, but suggested that EEG research can help find objective markers of vection (Palmisano et al., 2015). The current study, however, used a stimulus presentation of 1 s, which is too short to induce any sensation of vection (Palmisano et al., 2015), and had a static scene of 3 s in between every motion condition to avoid motion adaptation (Heinrich, 2007). In addition, we did not ask whether or not the participants were experiencing vection. This is necessary since vection is a (conscious) subjective sensation of self-motion (Palmisano et al., 2015), and participant report is crucial for establishing whether perception of vection has occurred. Further studies should use longer stimulus presentations and gather subjective reports of vection during the EEG recordings. This could determine whether or not changes in motion speed have any effect on perceived vection, and how this affects the N2 component of visual motion.

In addition to VEP analysis, a time-frequency analysis was carried out to study changes in brain oscillations in response to visual motion. In the TSE analysis the static control condition was compared with the three different speeds in the motion condition. The present findings showed alpha-band de-synchronizations in several parietal and occipital sources in response to visual motion. However, significant differences were mainly found between alpha-band synchronizations and de-synchronizations in the PM source. There were no significant differences between the three speeds of visual motion in the TSE analysis. This is probably because the same optic flow pattern was shown, only moving at three different speeds. So it may be that the motion sensitive areas of the brain are responding to visual motion, irrespective of speed. Differences lie in the perceived speeds of motion, not in the environment the participants find themselves in, and the differences between the three speeds are only seen in the VEP analysis of the N2 component. If the alpha de-synchronizations reflect the general processing of visual motion (Pfurtscheller et al., 1994), then VEP latency and amplitude reflect the processing time and load, respectively.

Occipital and parietal de-synchronizations in alpha-band frequencies are thought to reflect an activated state (Pfurtscheller et al., 1994; Klimesch, 1999), and this fits well with the present findings. There was a long alpha de-synchronization in the PM source in response to visual motion, followed by synchronization in response to the static condition, which is related to a deactivated or resting period. Pfurtscheller et al. (1994) reported two different alpha bands: a lower one reflecting visual processing situated in occipital areas, and a higher one reflecting cognitive processes and attention situated in parietal areas. The present participants' alpha de-synchronizations were, however, too variable and the data not accurate enough to identify whether they were higher or lower alpha waves. Coupled with the findings of visually evoked N2 components, the induced oscillations very likely indicate visual motion processing. These findings seem to be in line with several other studies (Pfurtscheller et al., 1994; Schürmann et al., 1997; Klimesch, 1999) reporting alpha de-synchronizations in connection with visual stimulation.

In conclusion, we demonstrated that for high speeds of simulated forward motion, peak latency increases whereas peak amplitude decreases with speed. This suggests that for driving speeds, lower speeds are perceived as less demanding, or that more experience with lower speeds results in shorter latencies and higher amplitudes. With fewer neurons attuned to higher visual speeds, the motion sensitive areas of the adult brain appear to be less attuned to relatively high motion speeds of up to 75 km/h. These findings have implications for road traffic safety with adult perceivers taking longer to respond to, and having fewer neurons specialized for, higher driving speeds. In addition, significant differences between alpha de-synchronizations in response to visual motion and alpha synchronizations in the static condition were found in the parietal midline (PM) source. We suggest that the alpha de-synchronizations reflect an activated state related to the visual processing of simulated forward motion, whereas the alpha synchronizations in response to the static condition reflect a deactivated resting period.

## Funding

This project has been financially supported by the Norwegian ExtraFoundation for Health and Rehabilitation through EXTRA funds.

### Conflict of interest statement

The authors declare that the research was conducted in the absence of any commercial or financial relationships that could be construed as a potential conflict of interest.
